# Hearing Loss Increases Inhibitory Effects of Prefrontal Cortex Stimulation on Sound Evoked Activity in Medial Geniculate Nucleus

**DOI:** 10.3389/fnsyn.2022.840368

**Published:** 2022-03-01

**Authors:** Chenae De Vis, Kristin M. Barry, Wilhelmina H. A. M. Mulders

**Affiliations:** School of Human Sciences, The University of Western Australia, Crawley, WA, Australia

**Keywords:** guinea pig, hearing loss, medial geniculate nucleus, prefrontal cortex, sensory gating, frontostriatal, electrophysiology

## Abstract

Sensory gating is the process whereby irrelevant sensory stimuli are inhibited on their way to higher cortical areas, allowing for focus on salient information. Sensory gating circuitry includes the thalamus as well as several cortical regions including the prefrontal cortex (PFC). Defective sensory gating has been implicated in a range of neurological disorders, including tinnitus, a phantom auditory perception strongly associated with cochlear trauma. Recently, we have shown in rats that functional connectivity between PFC and auditory thalamus, i.e., the medial geniculate nucleus (MGN), changes following cochlear trauma, showing an increased inhibitory effect from PFC activation on the spontaneous firing rate of MGN neurons. In this study, we further investigated this phenomenon using a guinea pig model, in order to demonstrate the validity of our finding beyond a single species and extend data to include data on sound evoked responses. Effects of PFC electrical stimulation on spontaneous and sound-evoked activity of single neurons in MGN were recorded in anaesthetised guinea pigs with normal hearing or hearing loss 2 weeks after acoustic trauma. No effect, inhibition and excitation were observed following PFC stimulation. The proportions of these effects were not different in animals with normal hearing and hearing loss but the magnitude of effect was. Indeed, hearing loss significantly increased the magnitude of inhibition for sound evoked responses, but not for spontaneous activity. The findings support previous observations that PFC can modulate MGN activity and that functional changes occur within this pathway after cochlear trauma. These data suggest hearing loss can alter sensory gating which may be a contributing factor toward tinnitus development.

## Introduction

Sensory gating is the process of inhibiting irrelevant neural stimuli from reaching higher cortical areas, allowing for attention to more relevant or salient sensory information (Cromwell and Atchley, 2015). Sensory gating requires activation of the frontoparietal attention network, which consists of several cortical regions including the prefrontal cortex (PFC) (Ptak, 2012), which is known to play an important role in a myriad of cognitive functions including attention, memory and executive function (Jobson et al., 2021). Another critical component of sensory gating circuitry is the thalamus, the obligatory relay en route to cortex for all sensory input, but olfactory (Saalmann et al., 2012; Halassa and Kastner, 2017). In agreement, multiple pathways from PFC to thalamus have been shown to exist (Mathiasen et al., 2021; Rios-Florez et al., 2021).

Defective sensory gating has been implicated in a range of neurological disorders. For example, dysregulation of the inhibitory circuitry in thalamus as well as thalamocortical hyperconnectivity have been proposed to be involved in the sensory over-responsiveness that is observed in individuals diagnosed with autism spectrum disorders (Iidaka et al., 2019; Wood et al., 2021). Reduced sensory gating has also been reported in patients diagnosed with schizophrenia (Freedman et al., 2020) and anxiety disorders (Storozheva et al., 2021).

Hearing loss has been shown to lead to reduced sensory gating of auditory information (Campbell et al., 2020; Chen et al., 2021). This is in line with a MRI study showing reduced functional connectivity between auditory thalamus and multiple other brain regions, including parts of PFC, in individuals with hearing loss (Xu et al., 2019). Abnormal sensory gating has also been suggested to be involved in the development of tinnitus (Rauschecker et al., 2010, 2015; De Ridder et al., 2015; Sedley et al., 2019), a phantom auditory perception that is strongly associated with hearing loss and/or damage to the cochlea (Baguley et al., 2013). Indeed, human studies show that individuals with hearing impairment and tinnitus display decreased auditory sensory gating which correlates with their tinnitus severity (Campbell et al., 2019) and others show reduced grey matter in PFC of tinnitus patients (Leaver et al., 2011, 2012, 2016).

The PFC has no direct projections to the auditory thalamus, the medial geniculate nucleus (MGN), but has indirect, multi-synaptic projections involving the thalamic reticular nucleus (TRN) (Nakajima et al., 2019), which provides strong GABAergic input to the MGN (Pinault, 2004). In agreement, in our laboratory we have demonstrated functional connectivity between PFC and MGN in rats (Barry et al., 2017). Moreover, a recent elegant study from Nakajima and co-workers demonstrated that the PFC indeed modulates attentional filtering in MGN *via* inhibitory thalamic reticular networks (Nakajima et al., 2019).

Previously, we have demonstrated in a rat model that trauma to the cochlea results in altered connectivity between PFC and MGN (Barry et al., 2021) showing enhancement of inhibitory effects of PFC electrical stimulation on the spontaneous firing rates of MGN neurons. This observation demonstrates that damage to the auditory periphery can cause functional changes to the sensory gating circuitry. In the present study, we further investigated this phenomenon using a guinea pig model, in order to demonstrate the validity of our finding beyond a single species. Furthermore, we also recorded the effects of PFC electrical stimulation on sound-evoked responses in MGN, in addition to effects on spontaneous activity.

## Materials and Methods

### Animals

Fifteen guinea pigs (Cavia porcellus, Hartley Tricolor) of either sex, weighing 200 to 300 g, were obtained from the University of Western Australia’s Preclinical Facility (PCF). Guinea pigs were kept under controlled conditions and were provided with appropriate access to food, water and shelter throughout the duration of the experiment. They were divided into an experimental group (*n* = 5) which underwent an acoustic trauma procedure and a control group (*n* = 10) that received a sham procedure. Ethics regarding experimental procedures were approved by the Animal Ethics Committee of the University of Western Australia.

### Recovery Experiment for Acoustic Trauma or Sham Procedure

Animals received a subcutaneous (s.c.) injection of atropine sulphate (0.05 mg/kg; 0.1 mL), followed by an intraperitoneal (i.p.) injection of Diazepam (Pamlin 5 mg/kg, 5 mg/mL diazepam). Then, guinea pigs received an intramuscular (i.m.) injection of Hypnorm (1 mL/kg; 0.315 mg/mL fentanyl citrate + 10 mg/mL fluanisone) to induce full surgical anaesthesia. Animals were shaved at the incision site and received a s.c. injection of Lignocaine (0.1 mL 1% solution). Once full depth of anaesthesia was obtained, animals were placed on a heating blanket in a soundproof room and mounted into hollow ear bars that allowed controlled acoustic stimuli to be delivered to the animal. The cochlea was exposed by a skin incision followed by a small opening in the bulla. An insulated silver wire recording electrode was positioned on the round window. Compound action potential (CAP) recordings were made to assess peripheral auditory thresholds between 4 and 24 kHz (2 kHz steps) (Johnstone et al., 1979). CAP recordings (32 averages per recording) were made in response to 10 ms tone bursts with repetition rate of 4/s.

When normal hearing was confirmed (Johnstone et al., 1979), a unilateral hearing loss was induced in the left ear in the experimental group by an acoustic trauma (continuous pure tone at 10 kHz and 124 dB SPL for 120 min). Plasticine was used to block the right ear. A half-inch condenser microphone driven in reverse was used as a speaker (Bruel and Kjaer, type 4134). A DIGI 96 soundcard connected to a digital/analog interface (ADI-9 DS, RME Intelligent Audio Solution, Haimhausen, Germany) and a custom-built software program (Neurosound MI Lloyd) was used to synthesise acoustic stimuli. This method of acoustic trauma is used routinely in our laboratory and causes a small, frequency restricted permanent hearing loss (Mulders and Robertson, 2009; Mulders et al., 2011). Sham animals received the same surgery and measurement of peripheral thresholds by CAP, but with no acoustic trauma. Animals received a top up of Hypnorm (one third of original dose) halfway the acoustic trauma. After the acoustic trauma, another CAP audiogram was recorded to determine the extent of immediate hearing loss, the incision was sutured, and the animals recovered for 2 weeks before the final experiment.

### Non-recovery Experiment for Thalamic Recordings With Prefrontal Cortex Stimulation

Two weeks after the recovery experiment, guinea pigs were administered a s.c. injection of atropine sulphate (0.05 mg/kg; 0.1 mL), followed by an i.p. injection of sodium pentobarbitone (30 mg/kg). Full surgical anaesthesia was then achieved by an i.m. injection of Hypnorm (initial dose 0.15 mL). Lignocaine (0.1 mL 1% solution) was injected s.c. at the incision site. Once full depth of anaesthesia was obtained, guinea pigs were placed on a heating blanket in the soundproof room. An electrocardiogram (ECG) was used to monitor the depth of anaesthesia throughout the experiment. Anaesthesia was maintained by half of the initial dose of sodium pentobarbitone every 2 h and a full dose of Hypnorm every hour. Guinea pigs were artificially ventilated by carbogen (95% oxygen and 5% carbon dioxide) through a tracheotomy. Animals were then positioned into hollow ear bars and as described in the recovery experiment (see section “Recovery Experiment for Acoustic Trauma or Sham Procedure”), a CAP audiogram was recorded from both the left and right ear to determine peripheral auditory thresholds.

Then the skull was exposed and using coordinates from a guinea pig atlas (Rapisarda and Bacchelli, 1977), position of the PFC and MGN were determined. A dental drill was used to perform two small craniotomies. A custom made (tungsten in glass) bipolar stimulating electrode was positioned in the PFC and a recording electrode [impedance < 1 MΩ at 1 kHz (Merrill and Ainsworth, 1972)] was lowered into the MGN. To minimise dehydration and limit movement of the brain, 5% agar in saline was used to cover the craniotomies. Ten minutes prior to recording, guinea pigs were administered an i.m. injection of Pancuronium Bromide (0.1 mL).

The stimulating electrode (Platinum-iridium, concentric bipolar electrode with a 2–3 μm tip, impedance 200 K, World Precision Instruments) was connected to an A-M Systems Isolated Pulse Stimulator (Model 2100). Neurosound software controlled the timing of electrical stimuli. Electrical stimulation was delivered as shock trains (50 ms duration) with a pulse duration of 0.5 ms and at a rate of 200 Hz. The intensity of the electrical current ranged from 100 μA to 1 mA. To increase the chance of observing an effect in the MGN, the current used to stimulate the PFC was initially applied at maximum (1 mA). The current was then decreased until threshold if the recording remained stable. Threshold was determined as the lowest possible current intensity that could elicit a response that was different to the neuron’s firing pattern.

Single neurons in the MGN were identified using pure tone and broadband noise stimuli as a search stimulus. Noise stimuli were delivered to the left ear while the right ear was blocked with plasticine. A tungsten in a glass recording electrode was used to record single neuron activity in the MGN of the thalamus (Merrill and Ainsworth, 1972). Entry into the right (contralateral to noise stimulus) MGN was verified by the presence of a robust cluster response to broadband noise stimulus (50 ms duration, 1 ms rise and fall times, sample rate of 96 kHz) (Cook et al., 2021; Zimdahl et al., 2021). Electrophysiological recordings of single neuron activity were recorded in MGN in response to stimulation of the right (ipsilateral to MGN) PFC.

When a single neuron was isolated, characteristic frequency (CF) and acoustic threshold at CF were determined as described previously (Mulders et al., 2011; Barry et al., 2015, 2019). Spontaneous firing rates (SFRs) were measured for 10 sec. Peristimulus time histograms (75–100 stimulus presentations) were constructed at CF at 20 dB above threshold to determine neuronal response. Then histograms (500 ms samples; 50–100 sweeps) were collected to determine the effect of PFC stimulation as follows. Firstly, effects of PFC stimulation were investigated on spontaneous firing, followed by effects on sound evoked activity (at 20 dB above neuronal threshold). If an effect was observed, current was decreased in small steps (200–300 μA) until threshold was reached. For analysis, changes in firing rate were determined by comparing the total spike counts with and without electrical stimulation of PFC.

Once recordings were finished, animals received an overdose of Lethabarb (Sodium Pentobarbitone 325 mg/mL; 0.3 mL i.p injection). Guinea pigs were transcardially perfused by methods outlined by previous studies (Barry et al., 2015, 2017). Brain tissue was collected and stored overnight in 4% paraformaldehyde in 0.1 M phosphate buffer (PB), followed by cryoprotection in 30% sucrose in PB for another 24 h. Brains were cut on a freezing microtome-cryostat into 60 μm sections. Sections were mounted onto slides and allowed to dry overnight. Once dried, slides were stained with Toluidine Blue and coverslipped. A Nikon Eclipse 8oi was used to perform light microscopy. This procedure verified the position of stimulating and recording electrode tracks within the PFC and MGN.

### Statistical Analysis

Multiple *t*-tests, corrected using Holm-Sidak method, were used to determine if sham or acoustic trauma had an effect on peripheral auditory thresholds and if hearing thresholds were different in the experimental and control group. A Mann-Whitney *U* Test was used to determine differences in spontaneous firing rates, characteristic frequencies (CF) and neural (acoustic) thresholds at CF between animals with normal hearing and hearing loss. To characterise the effect of PFC stimulation on single neuron MGN activity PSTHs without and with stimulation were compared using a paired *t*-test, with each time-bin as a separate variable. Proportions of neurons displaying no effect, inhibition or excitation were compared using a Chi-square analysis.

## Results

### Compound Action Potential (CAP) Audiograms

**FIGURE 1:**
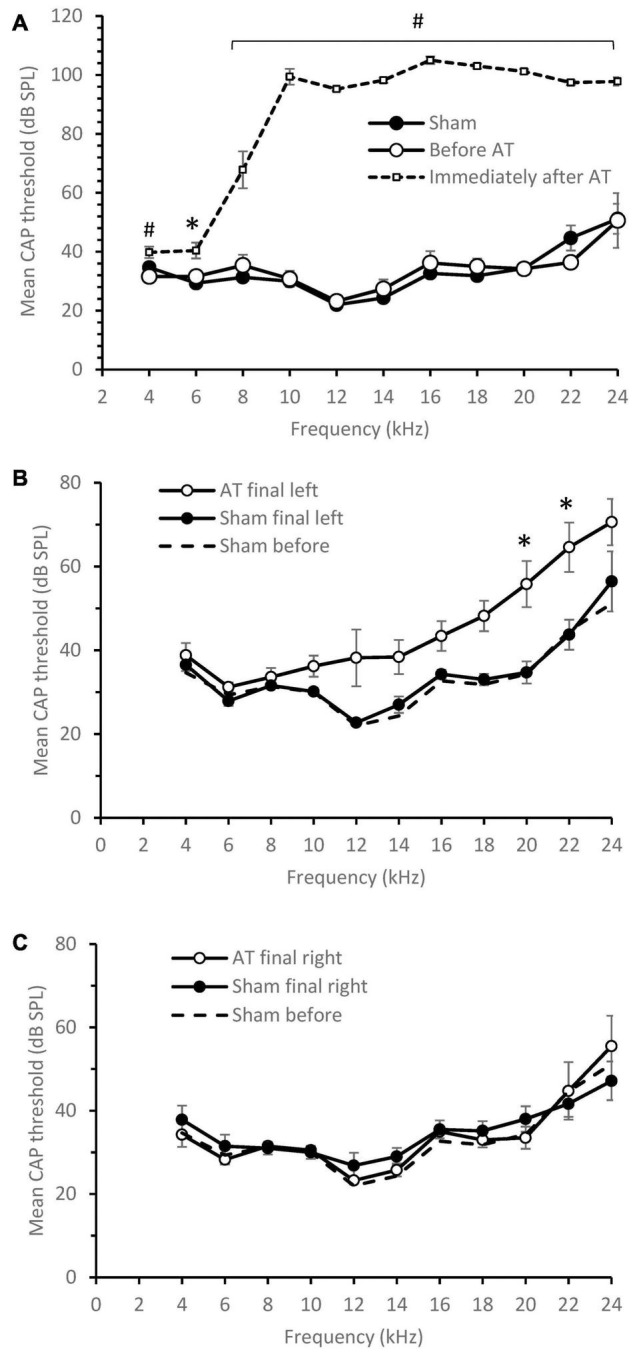
CAP threshold audiograms. **(A)** Mean CAP threshold left ear vs. frequency at the time of initial surgery, Sham animals (*n* = 6), AT animals before and after AT (*n* = 5). Significance between Immediately after and before AT indicated. **(B)** Mean CAP threshold left ear vs. frequency at the time of final surgery, Sham animals (*n* = 6), AT animals (*n* = 5). **(C)** Mean CAP threshold right ear vs. frequency at the time of final surgery, Sham animals (*n* = 6), AT animals (*n* = 4). For comparison the sham audiogram at initial surgery also plotted as a dotted line in **(B,C)**. Data Mean ± SEM. ^∗^*p* < 0.05, #*p* < 0.01.

The effects of acoustic trauma (AT) are shown in Figure 1. CAP thresholds in sham animals at the start of experiment were the same as in the AT group before the AT (Figure 1A). Immediately after AT was finished a significant increase in CAP thresholds was observed at all frequencies with the largest increase seen at and above the AT frequency (Figure 1A; multiple *t*-tests, corrected using Holm-Sidak method) in line with our previous publications (Mulders and Robertson, 2013; Mulders et al., 2016; Cook et al., 2021). After 2 weeks of recovery, at the time of the final experiment, CAP thresholds had partially recovered but a difference remained between sham and AT animals (Figure 1B). CAP thresholds showed a significance increase only at 20 and 22 kHz (*p* < 0.05), with threshold at 12 kHz approaching significance (*p* = 0.063, multiple *t*-tests, corrected using Holm-Sidak method). Right ears (not exposed to AT or sham surgery) showed similar thresholds at the final experiment (Figure 1C) as measured during initial surgery in the left ears of either group, confirming the absence of an effect of the AT on the contralateral side. In Figures 1B,C the original left ear thresholds of shams are plotted as well to illustrate the stable thresholds in the sham group and the normal thresholds in the right ear at the final surgery.

### Verification of Stimulating Electrode Position

**FIGURE 2:**
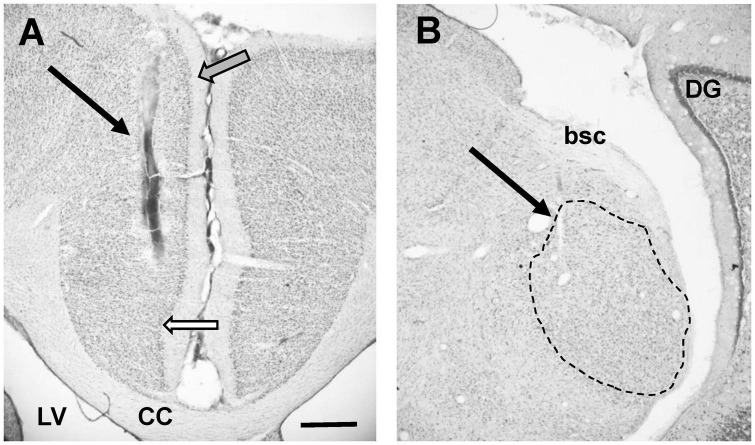
**(A)** Photomicrograph showing placement of stimulating electrode in PFC (arrow). Note that this image does not show deepest point of electrode. White filled arrow points to tentative border infralimbic and cingulate cortex. Grey filled arrow points at tentative border between cingulate cortex and secondary motor cortex. Please note that delineations of the cortex are based on rat atlases (Paxinos and Watson, 2007; Swanson, 2018) since the guinea pig atlas (Rapisarda and Bacchelli, 1977) does not show detailed delineations. **(B)** Photomicrograph showing placement of recording electrode in MGN (arrow). Dotted line shows outline of MGN, based on rat atlas (Swanson, 2018). Individual subdivisions of the MGN not indicated. CC, corpus callosum; LV, lateral ventricle; DG, dentate gyrus; bsc, brachium of the superior colliculus. Scale bar for both images: 0.5 mm.

Histology was used to verify the position of stimulating electrodes in all animals (Figure 2 showing an example). Five animals from the AT group and seven animals from the sham group showed placement of the stimulation electrode in the PFC (Rapisarda and Bacchelli, 1977). Only these animals showed effects on MGN recordings and were therefore included in further analyses. Electrode placement was explored in some animals by changing the depth of the electrode. Stimulation at a depth of approximately 5 mm from the cortical surface, most likely reflecting placement in prelimbic cortex (Hennessy et al., 2018), yielded the largest and most consistent effects on MGN activity.

### Medial Geniculate Nucleus (MGN) Neuronal Response Characteristics

Data were obtained from a total of 136 neurons with 68 neurons from control animals and 68 from AT animals. Characteristic frequency (CF) varied between 0.24 and 24.5 kHz (11.3 ± 0.8 kHz; mean ± SEM) in sham animals and between 0.6 and 19 kHz (8.1 ± 0.7 kHz; mean ± SEM) in AT animals. Mean CF was significantly different between the groups (unpaired *t*-test *p* = 0.017). It should be noted that CF could not be determined in seven neurons of the AT animals and four neurons of the sham animals though these neurons did show a response to noise. Mean SFRs were 0.66 ± 0.13 in AT animals (median 0.1) and 0.63 ± 0.15 in sham animals (median 0.1) and a Mann-Whitney showed no statistical difference (*p* = 0.899) between the groups.

**FIGURE 3:**
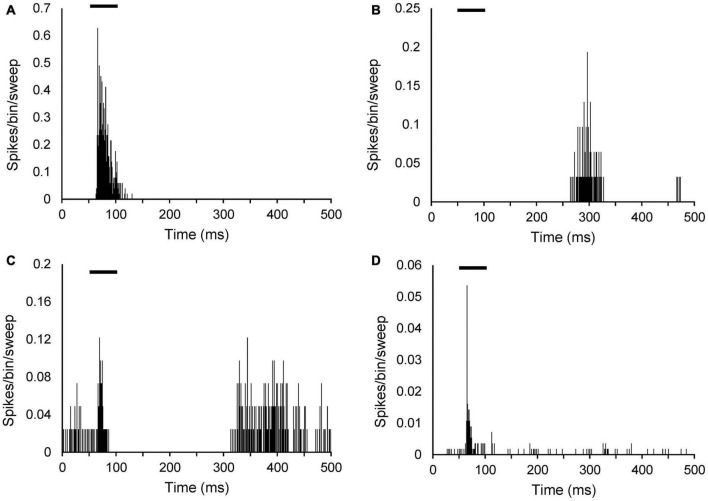
Peristimulus time histograms (PSTHs) illustrating different sound evoked response types in MGN neurons. PSTHs show single neuron responses to a 50-ms pure tone at CF 20 dB above threshold. Spikes are shown as per bin width (bin width of 1 ms) per sweep (50 sweeps). **(A)** Onset neuron (CF: 6.5 kHz; threshold 43 dB SPL). **(B)** Off response neuron (CF: 619 Hz; threshold 66 dB SPL). **(C)** On and off response neuron (CF 16.3 kHz; threshold 77 dB SPL). **(D)** Sustained response neuron (CF 15.6 kHz; threshold 41 dB SPL). Black bars in each panel indicate timing and duration of the tone burst.

Response type was based on a PSTH at CF 20 dB above threshold and could be determined in 57 of the neurons in AT animals and 61 of the neurons in sham animals. Examples of the PSTHs of the different response types are shown in Figure 3. Response types were in agreement with data described previous studies (Bordi and LeDoux, 1994; Barry et al., 2015, 2017). In both the sham and AT group, the majority of MGN neurons had an onset response to pure tone (46 neurons, 75.4% and 42 neurons, 73.7%, respectively), with a response latency ranging from 10 to 65 ms from the onset of the sound stimulus (Figure 3A). About a fifth of the neurons (12 neurons, 19.7% in sham group and 13 neurons, 22.8% in AT group) exhibited an on and off response (Figure 3C). An off response (firing related to offset of the tone burst) was observed in 1.6% (one neuron) of sham animals and 3.5% (two neurons) of AT animals (Figure 3B). The remainder of neurons exhibited a “sustained response” to pure tone (two neurons, 3.3% in sham animals, none in AT group) (Figure 3D). No difference was found in the proportion of response types between the sham and AT group [X2 (3, *N* = 118) = 2.422 *p* = 0.4895].

### Effects of Prefrontal Cortex Stimulation

Since inhibitory effects cannot be ascertained in neurons with a zero or very low SFR when using extracellular recordings, potential effects on SFRs were only investigated in neurons with a SFR > 0.4 spikes/sec. This meant that effects of PFC stimulation on SFRs were assessed in 26 neurons in AT animals and 28 neurons in sham animals. Mean CF was 10.35 ± 1.36 kHz (mean ± SEM) in sham animals and 7.0 ± 1.17 kHz in AT animals and this difference was not statistically different (unpaired *t*-test *p* = 0.0711). Note that in four of the sham animals and five of the AT animals CF could not be determined. In AT animals, excitatory effects were seen in three neurons (12%), inhibitory effects in eight neurons (31%) and no effect in 15 neurons (58%) (Note percentages rounded, so not adding up to 100%). In sham animals, excitatory effects were seen in four neurons (14%), inhibitory effects in 9 (32%) and the remaining 15 neurons showed no effect (54%). These proportions in type of effect were not different between the AT and sham animals [X2 (2, *N* = 54) = 0.1278 *p* = 0.9381].

The effects of PFC stimulation were also assessed on sound evoked activity (at CF, 20 dB above threshold) in 57 neurons from AT animals and 59 neurons from sham animals. Mean CF was 10.82 ± 0.83 kHz (mean ± SEM) in sham animals and 8.43 ± 0.69 kHz in AT animals and this difference was statistically different (unpaired *t*-test *p* = 0.0291). In one neuron of both sham and AT animals CF could not be determined. In AT animals, excitatory effects were seen in two neurons (4%), inhibitory effects in 21 neurons (37%) and no effect in 34 neurons (60%). In sham animals, excitatory effects were observed in one neuron only (2%), 17 neurons showed inhibitory effects (29%) and the remaining 41 neurons showed no effect (69%). Chi-square analysis showed again no significant difference between the prevalence of effect types between the AT and sham animals [X2 (2, *N* = 116) = 1.374 *p* = 0.5032].

Effects of PFC stimulation could be collected on both spontaneous and sound evoked activity from nine neurons in sham animals and 16 neurons in AT animals. From the nine neurons from sham animals, seven showed no effect on both spontaneous and sound evoked responses, one neuron showed excitatory effects on both and the remaining neuron inhibitory effects on both. From the 16 neurons from AT animals, five showed no effect on both responses and 4 neurons showed inhibitory effects on both. Four neurons showed an inhibitory effect on the sound evoked response without an effect on spontaneous firing, two neurons showed no effect on sound evoked responses but excitatory effects on spontaneous firing and the remaining neuron showed no effect on sound evoked responses but an inhibitory effect on spontaneous firing.

Although most neurons did not show an effect in response to PFC activation, it should be noted that they were located in the same tracks as neurons that displayed either inhibition or excitation. In addition, each animal included in the analysis showed some inhibitory or excitatory effects. It is therefore unlikely that the lack of effect was due to incorrect placement of the stimulating electrode. Threshold of stimulation, the lowest possible current intensity that could elicit a response, was collected in 29 neurons from AT animals and 18 neurons from sham animals. Mean threshold was 740 ± 46 μA in AT animals and 711 ± 59 μA and these values were not significantly different (unpaired *t*-test, *p* = 0.7221).

**FIGURE 4:**
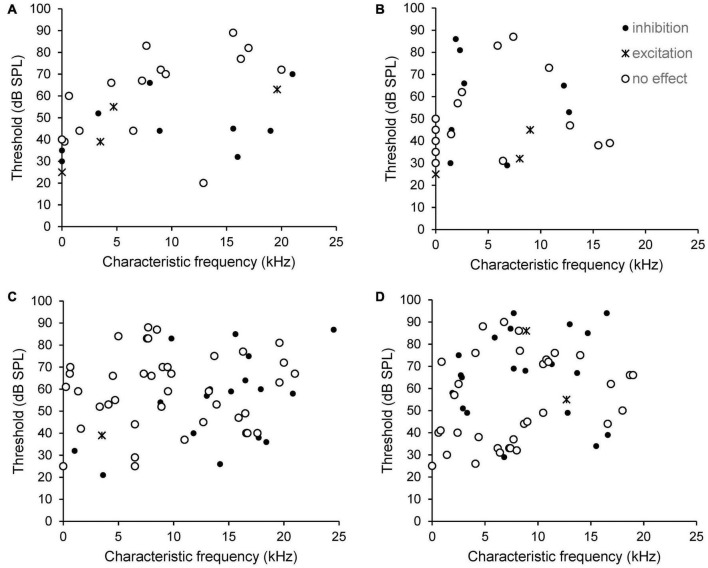
Scatterplots showing the relationship between CF, threshold and type of effect from electrical stimulation of the PFC on SFR (**A**, sham and **B**, AT) and on sound evoked responses at CF 20 dB above threshold (**C**, sham and **D**, AT). Neurons that could not be tuned to a pure tone are shown arbitrarily with a CF of zero and an arbitrary threshold, so appear on the *Y*-axis. Black dots: neurons showing inhibition. Crosses: neurons showing excitation. Open circles: neurons showing no effect.

**FIGURE 5:**
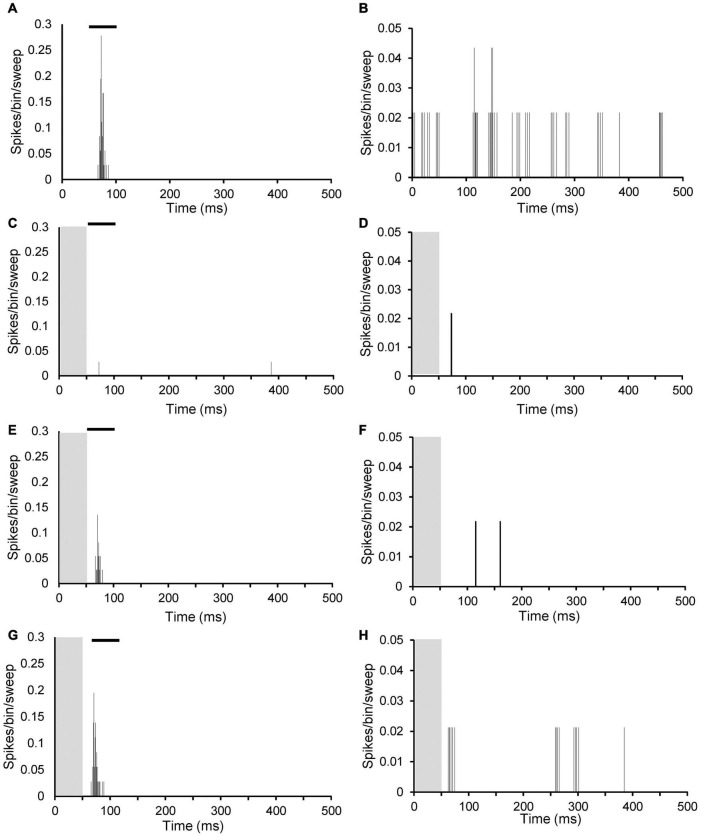
Peristimulus time histograms (PSTHs) illustrating inhibitory effects of PFC electrical stimulation at different stimulation strengths on sound evoked response at CF 20 dB above threshold (left column; **A,C,E,G**) and on spontaneous firing (right column; **B,D,F,H**). **(A,B)** No stimulation; **(C,D)** 1 mA; **(E,F)** 0.7 mA; and **(G,H)** 0.4 mA. Both neurons from an AT animal. Neuron left column CF 2.5 kHz with threshold 74 dB SPL and SFR 0.1 spikes/sec. Neuron right column CF 1.9 kHz with threshold 86 dB SPL and SFR 2.1 spikes/sec. Grey column indicates electrical stimulation and black bar in **(A,C,E,G)** illustrates timing of tone burst.

**FIGURE 6:**
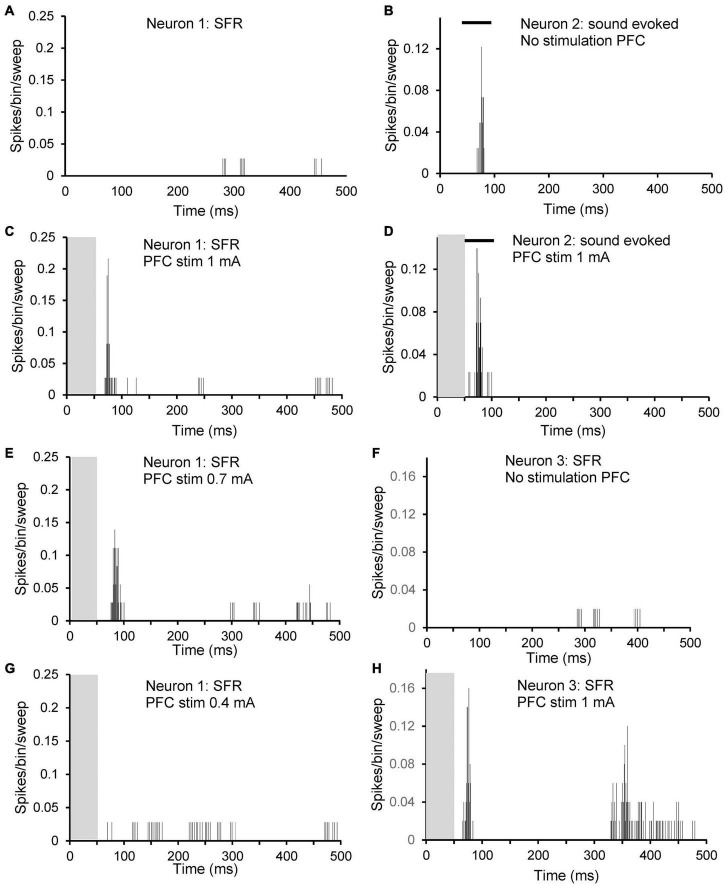
Peristimulus time histograms (PSTHs) illustrating excitatory effects of PFC electrical stimulation at different stimulation strengths on three different neurons. Neuron 1: **(A,C,E,G)**: spontaneous firing of a neuron with CF 1.9 kHz, threshold at 86 dB SPL and with SFR 2.1 spikes/sec. **(A)** No stimulation; **(C)** 1 mA; **(E)** 0.7 mA; and **(G)** 0.4 mA. Neuron 2: **(B,D)**: sound evoked firing at CF at 20 dB over threshold of a neuron with CF 8.9 kHz, threshold 86 dB SPL and with SFR of 0.2 spikes/sec. **(B)** No stimulation; **(D)** 1 mA. Neuron 3: **(F,H)**: spontaneous firing of a neuron with CF 4.7 kHz, threshold at 55 dB SPL and with SFR 0.4 spikes/sec. **(F)** No stimulation; **(H)** 1 mA. Note that neuron 1 showed no effect of electrical stimulation on sound evoked activity. Grey column indicates electrical stimulation and black bar in **(B,D)** illustrates timing of tone burst.

The type of effect from PFC stimulation, excitatory, inhibitory or no effect, did not correlate with CF or thresholds. This is shown in Figure 4. Data from SFR measurements are shown in panels 4A (sham) and B (AT) whereas effects on sound evoked firing rates are shown in panels 4C (sham) and D (AT). Please note that in this figure neurons that could not be tuned to a pure tone are shown arbitrarily with a CF of zero and an arbitrary threshold. Examples of inhibitory effects on sound evoked and spontaneous firing are shown in Figure 5. This figure also illustrates the effects of decreasing stimulation intensity. Examples of excitatory effects at different stimulation intensities are shown in Figure 6.

### Effect Size

**FIGURE 7:**
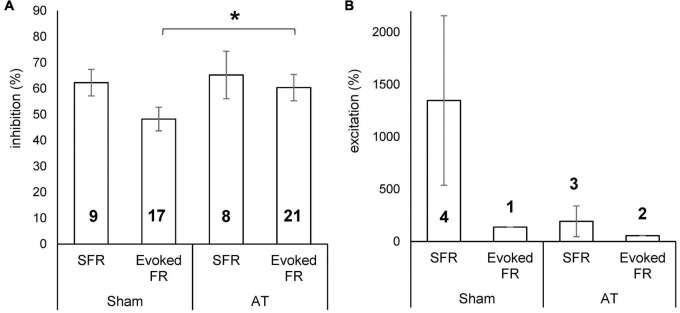
Bar diagrams showing percentage inhibition **(A)** and percentage excitation **(B)** on spontaneous firing rate (SFR) and sound evoked FR in sham and AT animals. Data Mean ± SEM. *p < 0.05. Numbers in bars represent number of neurons in which effect was demonstrated.

The magnitude of the effect from PFC stimulation was calculated as the percentage change from the PSTH of the unstimulated condition (spikes from 51 to 500 ms in PSTH to exclude contamination of stimulus artefact) and results are shown in Figure 7. The average inhibition in sham animals was 62 ± 5.1% (ranging from 41 to 83%) for SFRs and 48 ± 4.5% (ranging from 18 to 78%) for sound evoked responses. In AT animals the average inhibition for SFRs was 65 ± 9.2% (ranging from 35 to 100%) and for sound evoked responses 60 ± 5.1% (ranging from 24 to 100%) (Figure 7A). Excitatory effects on SFRs in sham animals ranged from 209 to 3740% (mean ± SEM, 1,348 ± 810). In the only neuron that showed an increase in sound evoked responses, the increase was 138%. In AT animals, excitatory effects on SFRs were seen in three neurons, ranging from 37 to 487% (193 ± 147%) and in two neurons in sound evoked response at 57 and 58% (Figure 7B). The magnitude of inhibition was significantly different between sham and AT animals with regards to the sound evoked response (unpaired *t*-test *p* = 0.045), but not for the SFRs. Due to the low numbers of neurons showing excitatory effects we did not run an analysis for statistical differences between groups.

**FIGURE 8:**
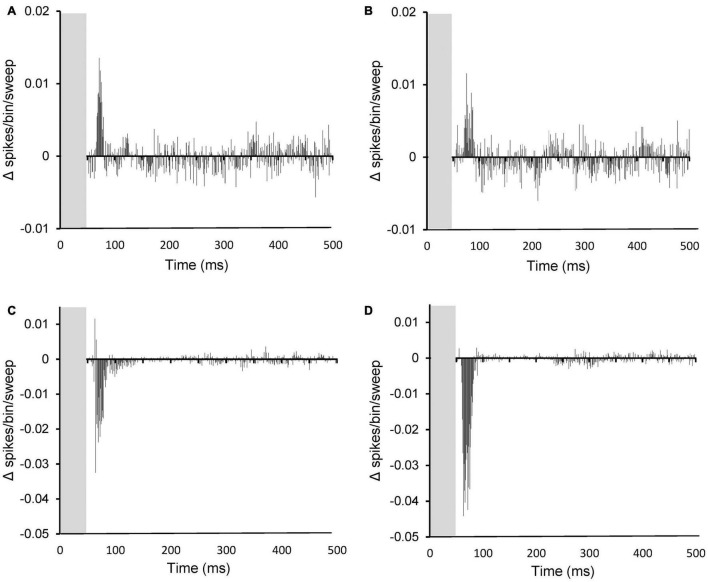
Peristimulus time histograms (PSTHs) showing the summed difference between PSTHs (per bin) with and without PFC stimulation on spontaneous activity **(A,B)** and on sound evoked activity **(C,D)**. **(A,C)** data from sham animals and **(B,D)**, data from AT animals. Note the increased inhibition after AT on sound evoked responses (compare panel **C** and **D**). **(A)** Based on 11 PSTHs, **(B)** on 26 PSTHs, **(C)** on 58 PSTHs, and **(D)** on 57 PSTHs.

To investigate the temporal pattern of effects of PFC stimulation, the average absolute magnitude of effect on SFRs and evoked FRs over time was calculated from the PSTHs (per bin) before and after PFC electrical stimulation for both sham and AT animals. Results are shown in Figure 8. The effects on SFRs are shown for sham (Figure 8A) and for AT animals (Figure 8B). In agreement with the findings above no apparent differences could be observed. The average effects on SFRs are dominated by the excitatory effects even though only few neurons showed an excitatory effect. This can be explained due to the general low SFRs, which means that even 100% inhibition is equivalent to only a small absolute change. The excitatory effects occurred within the first 50 ms after electrical stimulation had seized (grey column in graphs). The effects on sound evoked FRs are dominated by a large inhibitory effect in the first 50 ms after stimulation had stopped (Figure 8C, shams and 8D, AT animals). The significant increased inhibitory effect on sound evoked FRs in AT animals compared to shams is clearly apparent when comparing Figures 8C,D.

## Discussion

This paper provides the first evidence for a functional effect of PFC activation on both spontaneous and sound evoked responses in MGN using a guinea pig model. Electrical stimulation of PFC resulted in inhibitory effects in about one third of the MGN neurons and excitatory effects in about 5% of neurons, with no effect on the remainder of neurons recorded. This result supports the notion that the PFC may be involved in the sensory gating of auditory information. In addition, the data show that the magnitude of the inhibitory effects on sound evoked responses increased in animals exposed to an acoustic trauma that resulted in a small permanent hearing loss. This result suggests that noise induced hearing loss may alter the sensory gating of auditory information.

The present results regarding effects on SFRs in control animals are in broad agreement with previous studies in rats (Barry et al., 2017, 2021). Electrical stimulation of PFC resulted in either no effect, excitation or inhibition of MGN neuronal activity. However, whereas in the present study more than half of the neurons showed no effect of PFC stimulation, in the studies of Barry et al. (2017, 2021) no effect was only seen in about 20% of neurons. It should be noted that neurons that did not exhibit an effect were found in the same tracks as neurons that displayed either excitation or inhibition, which demonstrates that the lack of effect did not occur as a result of incorrect electrode placement or due to low levels of current intensity. Although it cannot be excluded that this represents a species difference, it is more likely due to the different methods used to classify the effects. Barry et al. (2017, 2019) used the criterion of >10% change in total number of spikes with and without stimulation to distinguish no effect from excitation or inhibition. In the present study, a paired *t*-test was used to compare between the histograms without and with stimulation. This analysis resulted in the smallest change being 35% on SFRs and 18% for sound evoked responses, which means that some smaller changes that were here classified as no effect, would have been incorporated as excitation or inhibition in the results of Barry et al. (2017, 2021), leading to a smaller proportion of neurons showing no effect in their data.

Similar to described in the rat (Barry et al., 2017, 2021), electrical stimulation of the prelimbic cortex resulted in the most pronounced and consistent results in MGN. Some of the functions associated with rodent prelimbic cortex, such as fear learning and attentional processes (Frysztak and Neafsey, 1991; Broersen and Uylings, 1999; Corcoran and Quirk, 2007) are in humans associated with activation of the ventromedial and dorsolateral PFC (Brown and Bowman, 2002; Uylings et al., 2003), suggesting some analogy between these structures in humans and rats. In addition, similar to rats and humans, the medial prefrontal cortex of the guinea pig has afferent and efferent connections with the mediodorsal nucleus of the thalamus (Markowitsch and Pritzel, 1981; Groenewegen, 1988; Uylings and van Eden, 1990).

The anatomical pathway by which PFC affects MGN activity is not yet fully elucidated. No evidence exists for a direct monosynaptic pathway and an indirect pathway *via* the thalamic reticular nucleus (TRN) is likely. The TRN, which provides predominantly GABA-ergic input to MGN (Yu et al., 2009a,b), receives indirect input from PFC (O’Donnell et al., 1997) and the GABA-ergic input would explain the predominantly inhibitory effects observed. Alternatively, other indirect pathways exist, which could account for the effects observed, potentially involving nucleus accumbens (O’Donnell et al., 1997). Past studies have proposed a multisynaptic network involving the nucleus accumbens as it is interconnected with both the PFC and the TRN (Sesack et al., 1989; O’Donnell et al., 1997; Rauschecker et al., 2010). Additionally, it has also been found that the nucleus accumbens projects indirectly to the TRN *via* the ventral pallidum and the globus pallidus (O’Donnell et al., 1997). Multisynaptic pathways may also involve or feedback connections from auditory cortical areas (Zhang and Suga, 2000; Winer et al., 2001; He et al., 2002), which receive direct input from PFC (Barbas et al., 2005; Golubic et al., 2014, 2019; Medalla and Barbas, 2014; Winkowski et al., 2018). The fact that different pathways may exist between PFC and MGN may explain the divergent effects (excitatory vs. inhibitory) observed in MGN following PFC stimulation.

Our observation that an AT and subsequent hearing loss increased the magnitude of the inhibitory effects observed in MGN, is in broad agreement with the recent study of Barry et al. (2021) in rats. However, the former study described this AT induced change on the effect on SFRs whereas in the present study it was only observable in the sound evoked FRs. Barry et al. (2021) did not investigate effects on sound evoked FRs. The fact that no change in effect magnitude on SFRs was observed in the present study may be due to the fact that SFRs were low as compared to other studies (Barry et al., 2019; Cook et al., 2021), which impacts the ability to measure an inhibitory change. For sound evoked responses this is not an issue as the evoked responses were generally robust and indeed the change in effect magnitude was clearly visible and statistically significant. The reason for the lower than expected SFRs may be the sampling of neurons. In previous studies aimed at investigating the average SFRs without and with AT (Kalappa et al., 2014; Barry et al., 2019; Cook et al., 2021), the emphasis is generally on collecting data from as many neurons as possible, and only a limited number of data are collected from each neuron. In the present study, the time taken for each neuron to obtain all required information, was longer and hence resulted in less neurons to be able to be recorded. This also means the possibility cannot be excluded that the populations of neurons recorded from is different as compared to our previous study in guinea pig (Cook et al., 2021). Finally, it is also possible that the difference between Barry and colleagues’ study in rat and our study in guinea pigs is due to intrinsic species differences.

Since the proportion of neurons showing inhibitory effects did not change after AT and subsequent hearing loss, this means it was the inhibitory effect on individual neurons that was increased. The inhibitory effect most likely involves GABA-ergic input from TRN (Yu et al., 2009a,b). The change observed may be due to either presynaptic or post-synaptic plasticity. Presynaptic GABA content and thereby GABAergic efficacy can be modified by alterations in the synthesis, transport, and degradation of GABA or related molecules (Roth and Draguhn, 2012). Post-synaptic changes may involve GABA receptor clustering (Marsden et al., 2007; Petrini et al., 2014), increasing the effect of GABA release on the post-synaptic MGN cell. This plasticity or synaptic scaling of the functional connection between PFC and MGN, may be driven by the hyperactivity that is induced by AT as described in a multitude of auditory structures including the MGN (Mulders and Robertson, 2009; Kalappa et al., 2014; Eggermont and Roberts, 2015; Wu et al., 2016; Cook et al., 2021).

As suggested by Barry et al. (2021) such a homeostatic mechanism, observed only 2 weeks after AT, may be an early compensatory effect to prevent the conscious perception of the hyperactivity that evolved due to AT. An increase in inhibitory effects from PFC activation, as observed in our study, is not in line with what has been suggested to occur in tinnitus development, which is thought to be due to a breakdown of inhibition (Rauschecker et al., 2010, 2015; De Ridder et al., 2011). So why do some individuals with hearing loss continue to develop tinnitus, and other do not (Baguley et al., 2013)? Is it possible that this compensatory mechanism fails in some individuals over time thereby allowing the hyperactivity to reach cortex and lead to a percept such as tinnitus? Populations with high levels of anxiety or post-traumatic stress disorder show higher prevalence of tinnitus (Hinton et al., 2006; Shargorodsky et al., 2010; Clifford et al., 2019). Anxiety is associated with both structural and functional changes in PFC (Hare and Duman, 2020), which may possibly affect the ability of the system to suppress tinnitus-like activity. In agreement with this notion, patients with panic disorder show reduced sensory gating as compared to healthy controls (Cheng et al., 2019). Further studies comparing the functionality of the PFC-MGN circuitry in animals with and without tinnitus are necessary to investigate the role of PFC in the development of tinnitus.

## Data Availability Statement

The raw data supporting the conclusions of this article will be made available by the authors, without undue reservation.

## Ethics Statement

The animal study was reviewed and approved by the Animal Ethics Committee of the University of Western Australia.

## Author Contributions

WM and CD contributed to conception and design of the study. CD, WM, and KB performed the statistical analysis and wrote sections of the manuscript. CD wrote the first draft of the manuscript. All authors contributed to manuscript revision, read, and approved the submitted version.

## Conflict of Interest

The authors declare that the research was conducted in the absence of any commercial or financial relationships that could be construed as a potential conflict of interest.

## Publisher’s Note

All claims expressed in this article are solely those of the authors and do not necessarily represent those of their affiliated organizations, or those of the publisher, the editors and the reviewers. Any product that may be evaluated in this article, or claim that may be made by its manufacturer, is not guaranteed or endorsed by the publisher.
